# Editorial

**Published:** 2013-09-06

**Authors:** Shibal Bhartiya, Tarek Shaarawy, Tanuj Dada

“This above all: To thine own self, be true.“

― William Shakespeare

Dear Friends,

It brings us great pleasure to bring to you this issue of the Journal of Current Glaucoma Practice.

This issue draws special attention to the three key elements for successful topical pharmacotherapy for glaucoma patients: adherence, persistence and correct administration.

Mohindroo et al ascertain the knowledge, attitude and practices followed by patients suffering from glaucoma and report that a broad variation exists in the reported practices, even in the very basic prerequisites of instilling eyedrops like washing of hands, checking the expiry date before the usage of eyedrops. The authors suggest a need to better educate our patients by providing them detailed information about eyedrop and its administration and also highlight many of the methodological problems.

Gupta et al elucidate compliance rates among glaucoma patients in a tertiary healthcare center, reasons for noncompliance and response-based-solutions to improve compliance in the same cohort. The authors reported that besides availability of medications at reasonable cost, simplification of treatment regimen and interactive health education appear to be the most important factors for improving compliance so that patients do not feel guilty or inadequate because they have problems while administering their eyedrops.

The effect of patient education on videotaped topical instillation of artificial tear drops on subsequent topical instillation is assessed by Lazcano-Gomez et al. They report that a single educational session on the proper use of topical drops improves the successful instillation of eyedrops. However, the authors also elaborate that they have not determined whether the patients will retain the improved instillation technique for long-term or if the intervention results in only a short-term improvement.

Juxtacanalicular trabecular meshwork and endothelial lining of Schlemm’s canal have been cited as the loci of aqueous outflow resistance, both in a normal as well as a glaucomatous eyes, and there has been a trend towards more physiological approach, with regard to resistance to aqueous outflow in newer surgical approaches. In the basic sciences section, Bhartiya et al elucidate the basis for currently available surgical modalities in light of the available histopathological evidence, regarding localization of outflow resistance.

Berezina et al report a case of successful repair of an exposed glaucoma drainage tube by cornea graft fixation with tissue adhesive, and without subsequent coverage by adjacent conjunctiva or donor tissues in a patient with history of keratoglobus with thin cornea and sclera, and phthisical contralateral eye.

We hope you find this issue relevant to your day-to-day clinical practice and look forward to your feedback.

Best wishes

**Shibal Bhartiya**

**Tarek Shaarawy**

**Tanuj Dada**

